# Effect of Haematological Parameters in the Development of Urethrocutaneous Fistula After Hypospadias Surgery

**DOI:** 10.7759/cureus.36033

**Published:** 2023-03-11

**Authors:** Kemal Gümüş, Mehmet Demir

**Affiliations:** 1 Urology, Mehmet Akif Inan Education and Research Hospital, Şanlıurfa, TUR; 2 Department of Urology, Harran University, Şanlıurfa, TUR

**Keywords:** platelet–lymphocyte ratio, neutrophil-lymphocyte ratio, haematological parameters, urethrocutaneous fistula, hypospadias

## Abstract

Investigation of the relationship between urethrocutaneous fistula (UCF) development and haematological parameters after hypospadias surgery was aimed for in this study. Patients who underwent tubularized incised plate urethroplasty between January 2015 and June 2021 with the diagnosis of distal hypospadias were included in the study. We divided the participants into two groups based on UCF development. We compared haematological parameters, including neutrophil, lymphocyte, and platelet counts; neutrophil-lymphocyte ratio (NLR); platelet-lymphocyte ratio (PLR); and systemic immune inflammation index (SII) values between the two groups. A total of 78 patients were included in the study. Of the patients, 11 developed UCF. The mean age of the patients was 74.9 ± 42.8 months. Catheter diameter, operation time, neutrophil counts, NLR, and SII values were similar between those with and without UCF (p > 0.05). However, the UCF group had significantly higher lymphocyte and platelet counts than those without UCF (p < 0.05). Moreover, the PLR value was significantly lower in the UCF group (p < 0.05). Patients who developed UCF post hypospadias surgery had a significant association with altered blood cell counts, including increased lymphocytes and decreased PLR rate. The PLR can be used as a biological marker for UCF development.

## Introduction

Hypospadias is a common congenital anomaly that affects approximately 1 in 300 male births. It occurs due to incomplete fusion of the urethral folds on the ventral side of the penis. Multifactorial etiology, including genetic, hormonal, vascular anomalies, and environmental factors, were suggested to play a role in hypospadias development [1].

Hypospadias is treated surgically, and the surgery aims to repair the urination and sexual function and appearance of the penis. Despite significant advances in the surgical treatment of hypospadias, complications occur in approximately one-quarter of cases [2]. The common complications include urethrocutaneous fistula (UCF), penile chord correction and glanular dehiscence, and meatal stenosis. Factors such as age at repair, hypospadias type, surgical technique, surgeon's experience, and the urethral tissue's healing capacity determine the operation's success [3].

The clinical significance of haematological parameters in wound healing remains unknown [4]. Post-surgery wound healing consists of hemostasis, inflammation, proliferation, and maturation stages. Platelets, leukocytes, macrophages, fibroblasts, endothelial cells, and molecules such as interferon, proteoglycans, integrins, matrix metalloproteinases, glycosaminoglycans, and other regulatory cytokines play critical roles during these stages [5]. A disruption in the wound healing stages might cause complications. Blood cell abnormality was implied to play a role in post-surgical complications. Therefore, we hypothesised that an abnormality in blood cell count might be linked to the hypospadias surgery complication of UCF.

The relationship between UCF and haematological parameters has not been studied before. We aimed to study post-surgical UCF development and neutrophil-lymphocyte ratio (NLR), platelet-lymphocyte ratio (PLR), and systemic immune inflammation index (SII) relationship.

## Materials and methods

Patients with distal hypospadias who underwent hypospadias surgery by a single specialist surgeon from January 2015 to June 2021 were included in the study. The institutional ethics committee of Harran University approved the study (HRU.22/11/11). The patients who underwent single hypospadias surgery and were followed up for at least six months were included in the study. The medical charts of participants were reviewed retrospectively. Those with more than one surgery for hypospadias, post-surgery infection, insufficient data on the chart, were excluded. Seventy-eight patients met the eligibility criteria.

The tubularized incised plate urethroplasty technique was used in the surgery. This method consisted of the following stages. After general anaesthesia, the meatus was re-evaluated and the surgical procedure was planned. A traction suture was placed on the glans penis. The penile skin was degloved up to the penoscrotal region. Artificial erection was induced by injection of sterile saline solution into the corpora cavernosa through a butterfly needle to determine the degree of deviation. The urethral plate was incised in the midline from the desired meatus point to the hypospadias meatus. After preparing the urethral plate, creating a tube over the catheter, the glandular wings were laterally dissected to cover the tension-free neourethra in the midline. Both sides of the urethral plate were sutured using 7/0 polydioxanone continuous sutures over a 6-8 Fr silicone urethral catheter according to the urethral calibration. The neourethra was covered with a vascularised dartos flap prepared from the subcutaneous tissue of the dorsal preputial skin or penile shaft. The dartos fascial flap was sufficiently mobilised to prevent penile torsion. The glandular wings and ventral skin defect were closed using 5/0 vicryl sutures. A standard midline closure was applied to the skin. These procedures were the same for all patients and performed by the same specialist surgeon. The patients were kept in the hospital for wound care until their catheters were removed and their regular dressings were applied. Catheters were removed on postoperative days five to seven. The patients were followed up on postoperative months one, three, and six.

The patients with and without UCF complications composed groups I and II, respectively. Blood samples were collected during anaesthesia preparation, and neutrophil, platelet and lymphocyte counts were analysed from the pre-operative hemogram results. NLR was calculated by dividing the neutrophil count by the lymphocyte count and PLR was calculated by dividing the platelet count by the lymphocyte count. SII was calculated by multiplying the neutrophil count by the platelet count and dividing the result by the lymphocyte count. Lymphocyte, neutrophil, and platelet counts, as well as NLR, PLR and SII were compared between the two groups.

Statistical analysis

Statistical Package for Social Sciences (SPSS) Version 28.0 (IBM Corp., Armonk, NY, USA) was used for data analysis. Mean, standard deviation, median (min-max), frequency, and ratio values were used in the descriptive statistical analysis of the data. Conformity to normal distribution was checked with the Kolmogorov-Smirnov test. The independent sample t-test and Mann-Whitney U test were used to analyse quantitative independent data. The chi-square test was used to analyse qualitative independent data, and the Fischer test was used when the chi-square test conditions were not met. The effect size and cut-off value were investigated using the receiver operating characteristic (ROC) curve.

## Results

A total of 78 patients were included in the study. The mean age of the patients was 74.9 ± 42.8 months. Of the patients, 11 developed UCF during the six-month follow-up (Table 1).

**Table 1:** Demographic data of patients NLR: neutrophil-lymhpocyte ratio, PLR: platelet-lymphocyte ratio, SII: systemic inflammation index

		Min - Max			Mean ± SD/n%		
Age (months)		14.0	-	151.0	74.9	±	42.8
Catheter size	6 fr				14		17.9%
	8 fr				64		82.1%
Duration of operation (min)		45.0	-	130.0	82.2	±	18.0
Neutrophil (×10^3^ /µL)		1.4	-	5.5	3.3	±	1.1
Lymphocyte (×10^3^ /µL)		1.7	-	9.3	4.0	±	1.8
Platelet (×10^3^/µL)		179.0	-	894.0	361.6	±	99.0
NLR		0.4	-	60.7	1.7	±	6.8
PLR		38.6	-	253.6	106.8	±	54.3
SII		112.0	-	1067.6	352.7	±	230.4
Urethrocutaneous fistula	No				67		85.9%
	Yes				11		14.1%

None of the patients in our study needed plication. Catheter diameter, duration of operation, neutrophil count, NLR and SII values were similar between the groups (p > 0.05). Patients with UCF had statistically significantly higher lymphocyte and platelet counts than patients without UCF (p < 0.05). PLR was significantly lower in patients with UCF (p < 0.05) (Table 2).

**Table 2:** Comparison of patients with and without urethrocutaneous fistula t: Independent samples t-test, m: Mann–Whitney U test, X²: Chi-square test (Fischer test). Statistically significant results are in bold italics (p < 0.05). NLR: neutrophil-lymphocyte ratio, PLR: platelet-lymphocyte ratio, SII: systemic inflammation index

		Urethrocutaneous fistula (−)					Urethrocutaneous fistula (+)				p	
		Mean ± SD/n%					Mean ± SD/n%					
Age (months)		70.1	±	42.6			104.3	±	31.9		0.009	m
Hypospadias level	Midpenile	25		37.30%			9		81.80%		0.006	X²
	Subcoronal	42		62.70%			2		18.20%			
Catheter size	6 fr	14		20.90%			0		0.00%		0.198	X²
	8 fr	53		79.10%			11		100.00%			
Duration of operation (min)		82.3	±	18.6			80	±	8.9		0.72	m
Neutrophil (×10^3^ /µL)		3.3	±	1.1			3.5	±	0.7		0.568	t
Lymphocyte (×10^3^ /µL)		3.8	±	1.6			6.8	±	1		0	m
Platelet (×10^3^/µL)		356	±	100.7			429.2	±	31.7		0.012	m
Hemoglobin (g/dl)		12.45	±	3.25			14.15	±	0.63		0.508	m
NLR		1.01	±	0.6			10.54	±	24.57		0.069	m
PLR		110.4	±	55			63.6	±	6.5		0.002	m
SII		363.6	±	236.5			221.7	±	30.1		0.177	m

**Figure 1:**
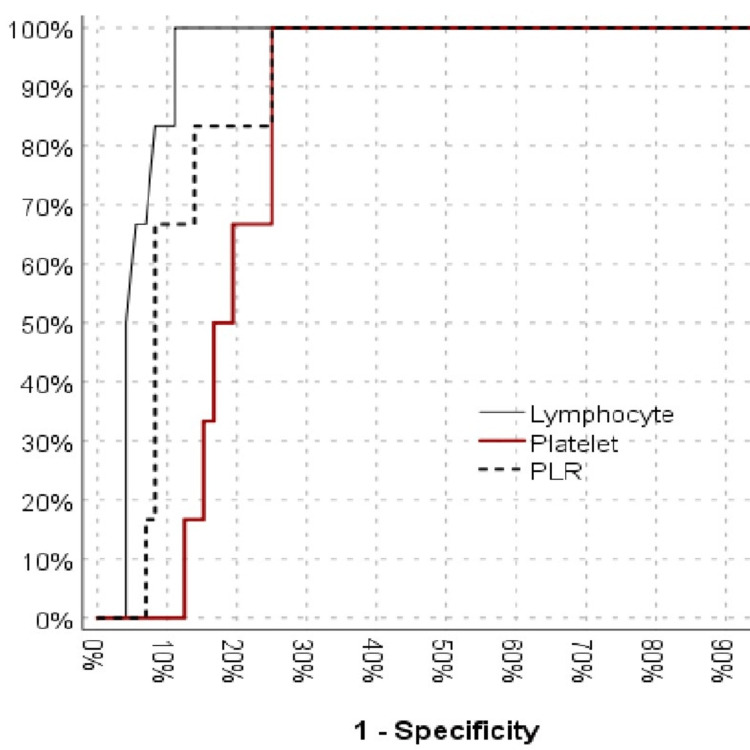
ROC curves of the derived blood parameters to urethrocutaneous fistula PLR: platelet-lymphocyte ratio, ROC: receiver operating characteristic

The UCF group had a significantly high lymphocyte number [area under the curve (AUC), 0.940 (0.886-0.993)]. The platelet counts successfully recognized patients with UCF [AUC, 0.810 (0.718-0.902)]. The PLR also had a significant impact on distinguishing between patients with and without UCF [AUC, 0.882 (0.800-0.964)] (Table 3). A PLR cut-off value of 75 had a sensitivity of 100%, a positive predictive value of 25%, a specificity of 75%, and a negative predictive value of 100% for the differentiation of patients with and without UCF (Figure 1).

**Table 3:** ROC analysis results of the derived blood parameters The cut-offs values were calculated by ROC analyses. Statistically significant results are in bold italics (p < 0.05). PLR: platelet-lymphocyte ratio, ROC: receiver operating characteristic

	Area under the curve	95% confidence interval			p
Lymphocyte (×10^3^ /µL)	0.94	0.886	-	0.993	0.001
Platelet (×10^3^/µL)	0.81	0.718	-	0.902	0.012
PLR	0.882	0.8	-	0.964	0.002

## Discussion

Complications after surgeries may occur regardless of the surgeon’s experience. Many surgical techniques have been developed to reduce complications and increase success rates. One of the most common complications of hypospadias surgery is UCF. The reported factors affecting UCF incidence include age, type of hypospadias, the tension in the suture lines, poor approximation of the neourethra, failure of the urethral catheter and infections [6]. However, UCF can develop for no particular reason. This has led researchers to investigate other factors. In recent years, inflammatory markers have played an important role in predicting the prognosis of malignancy, cardiovascular diseases, scar development in vesicoureteral reflux and rheumatic diseases [7,8]. In the present study, we focused on whether pre-operative haematological parameters affected UCF development.

Wound healing consists of hemostasis, inflammation, proliferation, maturation and remodelling stages. During the inflammatory stage, pathogens and foreign substances are removed from the injury, and the damage is brought under control. This phase begins immediately after the platelets achieve hemostasis. Vasodilation occurs, increasing vascular permeability and allowing neutrophils and monocytes to settle at the wound site. Cytokines and monocytes transform into macrophages in this phase [9]. In the proliferative phase, fibroblasts and collagen are formed. Angiogenesis occurs and nourishes the wound site. This is followed by a remodelling or maturation phase where the collagen type returns to normal [10]. These events show that neutrophils, lymphocytes and platelets play an active role in the wound healing. Following hypospadias surgery, an inflammatory response may lead to inadequate wound healing and UCF formation. Although nearly the same surgical methods are used in every patient and patients have the same risk factors, some patients develop UCF while others do not. It is also not always possible to pre-operatively predict which patients may develop a fistula. According to the hypothesis proposed in the present study, haematological parameters that play an active role in wound healing may also play an active role in fistula development after hypospadias surgery.

Coelho et al. examined pre-operative hemogram values in patients with acute extremity ischaemia and observed an increase in amputation and mortality in patients with high NLR values [11]. In a similar study, Vatankhah et al. reported better wound healing in diabetic wound ulcers in patients with low NLR [12]. Another study reported that the probability of spontaneous fistula closure increased as the NLR value decreased in patients who developed pancreatic fistula after distal pancreatectomy [13]. Topaktas et al. investigated the effect of NLR in predicting the recurrence of urethral stenosis after urethroplasty and reported that NLR was ineffective in predicting the recurrence of urethral stenosis [14]. In the present study, no difference was found in the NLR values of patients who developed UCF.

SII is a new inflammatory marker calculated from NLR and platelet count and used in cancer diagnosis and prognosis [15]. SII is proposed to be a better biomarker than NLR [16]. It has been widely used to reflect the immune status and determine some diseases prognosis and risk classification [17,18]. In the present study, although the SII value was lower in those with UCF, the difference was not statistically significant.

Lymphocytes and platelets are produced from the same haematopoietic stem cells. PLR should remain constant for hemostasis [19]. In the case of abnormal hematopoiesis, platelet count decreases more rapidly than lymphocyte count because the lifespan of the platelet is shorter, resulting in a decrease in PLR. Conditions that affect platelet production or lifespan can also affect wound healing. Therefore, PLR is critical in wound healing [20]. Maruyama et al. reported that surgical wound healing was poor when PLR values were lower [4]. Similarly, the present study found that patients with low PLR values developed UCF at a higher rate. In the present study, the probability of developing UCF increased as the lymphocyte and platelet counts increased, and PLR decreased. A PLR cut-off value of 75 had a sensitivity, specificity, positive predictive value and negative predictive value of 100%, 75%, 25% and 100%, respectively. This suggests that inflammatory cells operate in a balance and that a disruption in this balance impairs wound healing and leads to UCF.

One of the common complications requiring reoperation is UCF. These reoperations not only create a financial burden, but also cause anesthesia-related complications and long-term psychosexual problems [21]. For this reason, many treatment methods are tried to prevent the development of UCF. The use of intraoperative tissue adhesives has been shown to reduce the risk of UCF [22,23]. In another study, it was reported that additional coating of the neo-urethra with a double layer of dartos significantly reduced the rate of fistula after hypospadias [24]. On the other hand, there are studies reporting that the use of inlay grafts reduces the development of UCF and the risk of meatal/neourethral stenosis [25,26].

If the data in our study is supported by other studies and similar findings are reached, pre-op hematological parameters can be added to the risk factors for UCF. In patients with a high risk of developing UCF, additional measures such as tissue adhesives, inlay grafts, and double-layer grafts can be taken.

This study has some limitations. Due to the nature of the retrospective study, the collected data and the number of patients were limited. 

Other complications of hypospadias such as wound infection, meatal stenosis, glanular dehisence, penile chord may also be associated with hematological parameters. These complications were not evaluated in this study due to the small number of patients and the lack of long-term follow-up. 

Various parameters such as chordee grade, urethral plaque status and glans depth are effective in the formation of UCF, but these were not evaluated in our study.

The result only shows an overview of the association between hematological alteration and UCF, and we are unaware of any causal relationship. It is known that the complication rates of hypospadias surgery decrease as experience and the number of cases increase. In the present study, patients at the beginning and end of the case series were not compared among themselves.

## Conclusions

A simple blood count can help in determining the risk of UCF complications. Platelet and lymphocyte counts and PLR are practical, easily accessible, low-cost parameters already checked as part of the routine workup. In addition to the other UCF risk factors, checking these haematological parameters can give us a better idea of UCF development. In patients with a high risk of developing UCF, additional measures such as tissue adhesives, inlay grafts, and double-layer grafts can be taken. Further prospective studies involving multiple centres and large patient series examining long-term results are needed to validate these results.
